# Increased Plasma Dipeptidyl Peptidase-4 (DPP4) Activity Is an Obesity-Independent Parameter for Glycemic Deregulation in Type 2 Diabetes Patients

**DOI:** 10.3389/fendo.2019.00505

**Published:** 2019-07-25

**Authors:** Jit Sarkar, Titli Nargis, Om Tantia, Sujoy Ghosh, Partha Chakrabarti

**Affiliations:** ^1^Division of Cell Biology and Physiology, Indian Institute of Chemical Biology (CSIR), Kolkata, India; ^2^Academy of Scientific and Innovative Research, Ghaziabad, India; ^3^Community Health Program, SWANIRVAR, North 24 Parganas, India; ^4^Department of Minimal Access & Bariatric Surgery, ILS Hospitals, Kolkata, India; ^5^Department of Endocrinology and Metabolism, Institute of Postgraduate Medical Education and Research, Kolkata, India

**Keywords:** type 2 diabetes, visceral adipose tissue, DPP4, non-obese, obese

## Abstract

**Background:** Increase in circulating dipeptidyl peptidase-4 (DPP4) activity and levels has been reported to associate both with hyperglycemia and obesity. Here we aim to decipher the role of enhanced plasma DPP4 activity in obese type 2 diabetes (T2DM) patients.

**Materials and methods:** Plasma DPP4 levels and activity were measured in obese and non-obese newly diagnosed T2DM patients (*n* = 123). Visceral and subcutaneous adipose tissue DPP4 expression and activity were determined in 43 obese subjects (T2DM = 21 and non-T2DM = 22). 20 subjects undergoing Mini-Gastric Bypass (MGB) surgery were followed up over 4–6 weeks for plasma DPP4.

**Results:** Plasma DPP4 levels and activity both were increased in T2DM patients compared to control group. However, DPP4 levels and not DPP4 activity were increased in obese T2DM patients compared to non-obese T2DM (62.49 ± 26.27 μg/ml vs. 48.4 ± 30.98 μg/ml, respectively, *p* = 0.028). DPP4 activity in visceral adipose tissue (VAT) from obese T2DM and obese non-T2DM groups were similar (5.05 ± 3.96 nmol/min/ml vs. 5.83 ± 4.13 nmol/min/ml respectively, *p* = 0.548) in spite of having increased DPP4 expression in the obese T2DM group. Moreover, in obese patients, plasma DPP4 levels and activity did not show any significant change after weight reduction and glycemic control following MGB surgery.

**Conclusion:** Enhanced plasma DPP4 activity in T2DM occurs independently of obesity. Thus, adipose derived DPP4 may not be playing any significant role in glycemic deregulation in obese T2DM patients.

## Introduction

Dipeptidyl peptidase-4 (DPP4/CD26) is a widely expressed single pass type II transmembrane protein with a very short cytosolic tail and having a unique exopeptidase activity (1). The larger extracellular portion having the catalytic domain is cleaved off putatively by matrix mellatoproteases and released into the circulation as soluble DPP4 (sDPP4) whose level and enzymatic activity can be measured (2, 3). DPP4 cleaves N-terminal dipeptides from a variety of substrates including incretin hormones glucose-dependent insulinotropic peptide (GIP) and glucagon-like peptide-1 (GLP-1) and thereby impairs insulin secretion from pancreatic beta cells (4). Incretin hormones, particularly GLP-1 also controls food intake and overall body weight (5). DPP4 inhibitors are thereby clinically used as “incretinergic” drugs for type 2 diabetes mellitus (T2DM) (6).

Growing interest of DPP4 inhibitors as an anti-diabetic therapeutic owing to its cardio-protective effects (7) has led to the investigation of its plasma activity, plasma concentrations and its association with glycemic deregulation in T2DM by multiple groups. Interestingly, plasma DPP4 activity was reported to be both decreased (8, 9) and increased (10–12) in T2DM patients in the literature. However, most of the recent studies support the association of enhanced plasma DPP4 activity and/or levels to T2DM. But obesity being an important risk factor for T2DM and DPP4 being an adipokine, the role of adipose derived DPP4 in the pathogenesis of T2DM still remain debatable. Plasma DPP4 activity positively correlated with chronic hyperglycemia in both type 1 and T2DM patients (10–14) and served as an important predictor for the onset of insulin resistance both in type 1 diabetes (15) and T2DM (13). From the therapeutic aspect, DPP4 inhibitors have been introduced in the market from 2006 (16) and quite interestingly several randomized control trials (RCT) have reported better glycemic control achieved by these drugs specifically for the Asian population where there is a large population of non-obese T2DM (17, 18).

In the context of obesity, there has been a growing interest in the role of DPP4 in adipose tissue biology since its discovery as a novel adipokine (19). DPP4 expression is increased during adipose tissue differentiation with increased rate of secretion from adipose tissue in obesity and associated with body mass index (BMI) (20). In addition, DPP4 expression varies between adipose depot specific manner, visceral adipose tissue (VAT) depot being the highest expressor (19, 21). In agreement with these observations, plasma DPP4 enzyme activity was shown to be positively correlated with BMI in young healthy Japanese subjects (22) while serum DPP4 protein levels correlated with BMI in healthy South Asian and in obese insulin-resistant Caucasian population (23).

As obesity is one of the most important risk factors for the pathogenesis of T2DM (24), it may be possible that adipose associated increase in plasma DPP4 plays an important role in linking obesity to T2DM. But reports mentioning DPP4 activity not being at par with DPP4 levels (25, 26) owing to its structural heterogeneity pose further questions on whether DPP4 levels or activity is important in glycemic control. Using two different cohorts, we aim to investigate the relative contribution of adipose derived DPP4 to plasma DPP4 activity and hence its role in glycemic deregulation.

## Materials and Methods

### Patient Recruitment

In the first study cohort, 123 newly diagnosed T2DM patients (M = 49 & F = 74) were recruited from the Department of Endocrinology & Metabolism of Institute of Postgraduate Medical Education and Research (IPGME&R), Kolkata. 74 healthy controls (M = 39 & F = 35) were recruited from a community based health screening program run by a not-for-profit organization, SWANIRVAR. A criterion for T2DM was set as per the American Diabetes Association (ADA) (27). All the subjects were subdivided into obese and non-obese groups based on BMI < 25 (28, 29). The study was approved by human ethics committee of IPGME&R hospital and all subjects gave written informed consent.

In the second study cohort, 63 obese subjects (T2DM = 27; non-T2DM = 36) were recruited from the Department of Surgery, ILS Hospitals, Saltlake. Both subcutaneous adipose tissue (SAT) and VAT were collected from 43 (T2DM = 23; non-T2DM = 20) patients during sleeve gastrectomy, mini-gastric bypass (MGB), herniectomy, or cholecystectomy surgeries. Twenty subjects (T2DM = 4; non-T2DM = 16) from this group undergoing Mini Gastric Bypass (MGB) bariatric surgery were subsequently followed up over 4–6 weeks in whom pre-operative and post-operative blood samples and anthropometric measurements were taken. This study was approved by human ethics committee of ILS Hospitals, Saltlake and all the subjects gave written informed consent.

### Sample Collection and Anthropometric Measurements

All blood samples were collected in Sodium Fluoride/Na_2_ EDTA vials (BD Vacutainer, NJ, USA). Plasma was separated and stored at −80°C for long term storage. Anthropometric measurements were taken during sample collection. Weight, height, and waist circumference were measured. For SAT and VAT collection, the tissue was collected during the surgery and immediately suspended in RNA later solution so as to fully immerse the tissue. It was then collected to the laboratory for storage at −80°C.

### Biochemical Measurements

Plasma was used for biochemical measurements with reagents from Randox Laboratories Ltd. (County Antrim, UK). Plasma Glucose was measured by Glucose Oxidase method, Total Cholesterol by Cholesterol Oxidase method, Total Triglycerides by Lipase/GPO-PAP method. Glycated hemoglobin (HbA1c) was measured by HPLC (D10 Hemoglobin analyzer, Bio-Rad, Hercules, CA, USA). Plasma Leptin (RayBiotech, Norcross, GA, USA), Insulin (Merck Millipore, MA, USA), DPP4 (R&D Systems, MN, USA), and GLP-1(Active) (Merck Millipore, MA, USA) levels were measured by ELISA. Homeostatic model assessment (HOMA2) designed by Diabetes Trials Unit, The Oxford Center for Diabetes, Endocrinology, and Metabolism was used to estimate insulin resistance (HOMA2 IR) from all fasting venous samples.

### Gene Expression Analysis

Total cellular RNA was isolated from 50 to 100 mg homogenized adipose tissue samples using TRIzol reagent (Invitrogen). cDNA was synthesized from 1,000 ng of total RNA using cDNA synthesis kit (Roche). *DPP4* gene expression was analyzed by quantitative PCR (LightCycler 96 real time PCR, Roche) using SYBR Green master mix (FastStart Universal SYBR Green Master, Roche) with following primers—forward 5′AAGTGGCGTGTTCAAGTGTG3′ and reverse 5′GGCTTTGGAGATCTGAGCTG3′. Relative gene expression was analyzed by ΔΔCt method and normalized by 18S RNA.

### Immunoblotting

VAT and SAT samples were homogenized in cell lysis buffer (50 mM TrisHCl pH 7.4, 100 mM NaCl, 1 mM EDTA, 1 mM EGTA) containing 1% Triton X100 and protease inhibitor cocktail (Roche). Equal amount of total tissue lysates were separated by SDS-PAGE, transferred into Immobilon-P PVDF membrane (Millipore, Bangalore, India), and were probed with primary antibodies against CD26 (DPP4) (Abcam, Cambridge, UK) and β-actin (Sigma) followed by HRP tagged secondary antibody (Genei, Bangalore, India). DPP4 expression was visualized by enhanced chemiluminescence using LuminataClassico Western HRP substrate (Millipore, St. Charles, MO USA) and band intensity was normalized with β-actin using NIH Image J software.

### DPP4 Enzyme Assay

DPP4 activity in plasma and in tissue lysates was assayed as described earlier (3). Briefly, DPP4 activity was determined as the rate of 7-amino-4-methylcoumarin (AMC) cleavage per minute per mL from the synthetic substrate H-glycyl-prolyl-AMC (Sigma Aldrich, St. Louis, MO, USA). AMC fluorescence (excitation/emission-380/460 nm) was measured in a plate reader (Synergy H1 multi-mode microplate reader; Biotek, Winooski, VT, USA).

### Statistical Analysis

Statistical analysis was performed in RStudio (Version 1.1.447) and data are represented in GraphPad Prism 5 software (La Jolla, CA, USA). Descriptive summary of the data have been represented by mean and standard deviation. 95% confidence interval has been presented where relevant. Shapiro-Wilk's W test was performed to assess normality. Numerical variable have been compared between groups by independent-samples *t*-test or Mann-Whitney U test as appropriate. For paired analysis, paired *t*-test was used. Pearson's correlation coefficient “r” has been calculated to explore association between variables. Power was calculated at the significance level of 0.05 for the difference in DPP4 level between obese and non-obese T2DM groups. Sex adjustment was done using *lsmeans* package in R. Correlation coefficients between all the parameters was calculated by *Hmsic* package in R. *p* < 0.05 was considered to be statistically significant.

## Results

### Plasma DPP4 Activity Was Similar in Non-obese and Obese T2DM Patients in Spite of Increased DPP4 Levels in the Obese T2DM Group

Fasting plasma DPP4 levels, DPP4 activity, insulin and glucose concentrations were measured in newly diagnosed T2DM patients (*n* = 123) and BMI matched control subjects (*n* = 74). The mean values of different clinical and biochemical parameters in T2DM and non-diabetic subjects are depicted in Table 1. Consistent with previously published reports, both plasma DPP4 concentrations and activity were significantly increased in T2DM patients compared to control subjects irrespective of BMI distribution (Supplementary Figures 1A,B). To evaluate the relative contribution of plasma DPP4 in obesity and hyperglycemia, we divided the T2DM population into obese (*n* = 52) and non-obese (*n* = 71) sub-groups (Table 2). Although we found considerably higher plasma DPP4 levels in obese T2DM patients (Supplementary Figure 1C), but we did not find any difference in DPP4 activity between these groups (Supplementary Figure 1D). There was no difference in DPP4 levels between males and females within non-obese (44.7 ± 28.9 vs. 52 ± 33, *p* = 0.405) and obese (66.8 ± 29.8 vs. 60.9 ± 25.4, *p* = 0.511) T2DM groups. DPP4 levels were higher in the obese group than the non-obese group even after adjusting for sex (*p* = 0.037). Higher DPP4 level in the obese T2DM group had a power of 0.85 after considering a significance level of 0.05. Quite interestingly, DPP4 activity and DPP4 levels correlated significantly only in the non-obese T2DM group with no such correlation in the obese group (Supplementary Figures 1E,F). Paired correlation between all other parameters has been done for the non-obese and obese T2DM group separately and mentioned in Supplementary Table 1.

**Table 1:** The subject characteristics of healthy and T2DM patients and biochemical parameters.

	**Healthy**	**T2DM**	***P*-value**
N (male/female)	74 (39/35)	123 (49/74)	
Age (years)	39.34 ± 9.92	45.8 ± 8.13	<0.001
BMI (kg/m^2^)	27.34 ± 8.82	25.6 ± 6.04	0.62
WC (waist circumference) (cms)	97.43 ± 23.84	93.89 ± 15.3	0.746
FBS (fasting blood glucose) (mg/dl)	88.92 ± 10.22	162.58 ± 49	<0.001
HOMA2 IR	1.41 ± 0.91	1.39 ± 1.33	0.998
HOMA2 %B	118 ± 56	47.4 ± 37.4	<0.001
TG (triglycerides) (mg/dl)	135.49 ± 73.89	156.67 ± 89.91	0.0847
TC (total cholesterol) (mg/dl)	173.28 ± 39.89	187.87 ± 48.65	0.032
DPP4 activity (nmol/min/ml)	17.24 ± 12.09	22.34 ± 15.53	0.009
DPP4 concentration (μg/ml)	41.78 ± 30.01	53.38 ± 30.01	0.017

*Data represented by means ± SD. Healthy subjects are individuals without T2DM. p ≤ 0.05 considered statistically significant*.

**Table 2:** The biochemical parameters of non-obese and obese T2DM patients.

	**Non-obese**	**Obese**	***P*-value**
N (male/female)	71 (32/39)	52 (17/35)	
Age (years)	46.8 ± 8.91	44.42 ± 6.78	0.085
BMI (kg/m^2^)	22.05 ± 2.1	30.46 ± 6.3	<0.001
WC (waist circumference) (cms)	86.16 ± 7.53	104.99 ± 16.81	<0.001
FBS (fasting blood glucose) (mg/dl)	168.42 ± 45.83	154.6 ± 52.42	0.014
HOMA2 IR	1.18 ± 0.75	1.56 ± 0.96	0.084
HOMA2 %B	36.5 ± 21.4	56.1 ± 43.3	0.022
TG (triglycerides) (mg/dl)	165.84 ± 103.23	143.5 ± 65.18	0.4
TC (total cholesterol) (mg/dl)	181.07 ± 51.56	197.63 ± 42.82	0.067
DPP4 activity (nmol/min/ml)	21.2 ± 13.73	23.81 ± 17.62	0.794
DPP4 concentration (μg/ml)	48.4 ± 30.98	62.49 ± 26.27	0.028

*Data represented by means ± SD. Non-obese are individuals with BMI ≤ 25. p ≤ 0.05 considered statistically significant*.

### Adipose Tissue DPP4 Activity Is Not Related to Hyperglycemia in Obese T2DM Patients

**Figure 1:**
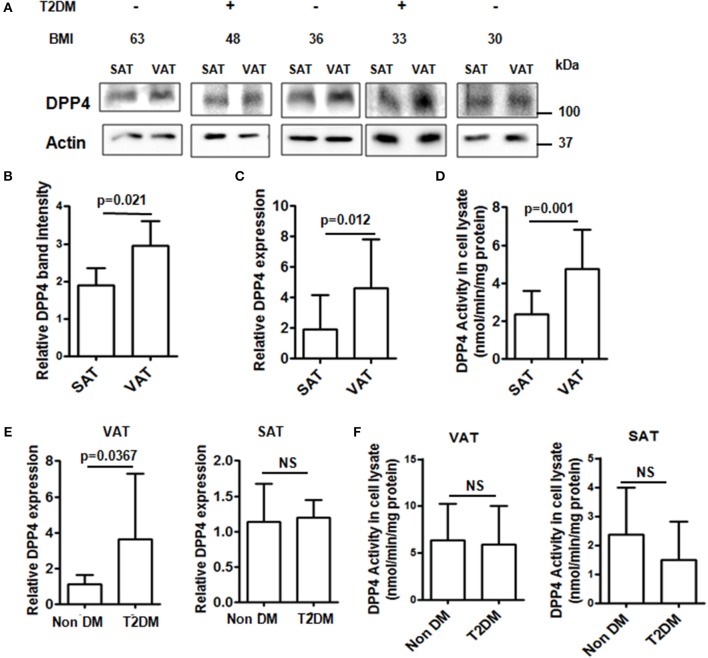
DPP4 protein levels, activity and expression in adipose tissue from obese patients. **(A)** DPP4 protein levels in SAT and VAT were analyzed by Western blot (*N* = 13). **(B)** Densitometric analysis of DPP4 protein levels normalized to actin. **(C)** Relative DPP4 gene expressions of SAT and VAT tissue. **(D)** DPP4 activity in SAT and VAT homogenate. **(E,F)** Adipose samples from T2DM (*N* = 20) and non-diabetic (*N* = 23) patients were analyzed for DPP4 activity and relative gene expression. **(A-F)** Data are represented as mean ± SD.

Obesity is tightly linked with the pathogenesis of insulin resistance and T2DM and augmented release of DPP4 from adipose tissue is associated with visceral obesity and insulin resistance. We therefore assessed DPP4 gene expressions, protein levels and enzymatic activities in the VAT and SAT depots from obese patients undergoing abdominal surgery with (*n* = 20) or without T2DM (*n* = 23) with comparable BMI (38 ± 11 vs. 42 ± 10, *p* = 0.224) and leptin levels (36147.75 ± 33624.77 pg/ml vs. 24707.27 ± 29473.27 pg/ml, *p* = 0.297). In agreement with previously published results (21), DPP4 protein levels, gene expressions and activity were significantly increased in VAT compared to SAT (Figures 1A–D). Next, we analyzed the same parameters in the fat depot between non-diabetic and T2DM groups. Though *DPP4* gene expression was higher in VAT of T2DM patients (Figure 1E) we did not find any difference in either VAT or SAT DPP4 enzymatic activity (Figure 1F) between these two groups indicating that adipose tissue derived DPP4 has no significant role in hyperglycemia.

### Glycemic Control and Decrease in BMI Does Not Accompany a Reduction in Plasma DPP4 Activity and Level in Patients Undergoing Mini-Gastric Bypass Surgery

To further confirm the hypothesis that plasma DPP4 activity is independent of obesity, we conducted a follow-up study with 20 obese patients (T2DM = 4 and non-T2DM = 16) undergoing MGB. Fasting plasma samples were collected before and after 4–6 weeks of the surgery and several biochemical assays were performed thereafter and are shown in Table 3. Although there was a significant difference in reduction of BMI (*p* < 0.001), FBS (*p* = 0.004), and insulin resistance (*p* = 0.019) before and after the surgery (Table 3 and Supplementary Figures 2A,B), we did not find any corresponding difference in plasma DPP4 activity (*p* = 0.084) and levels (*p* = 0.163) (Table 3 and Supplementary Figures 2C,D). Note that despite loss of significant weight patients were still within the obesity category after the surgery (BMI = 39.77 ± 5.13). Taken together our data suggest that glycemic control contributed by weight reduction does not accompany any reduction of plasma DPP4 activity. Indeed plasma DPP4 activity revealed an increasing trend after weight reduction although it did not reach statistical significance.

**Table 3:** The biochemical parameters of pre- and post MGB surgery patients.

	**Pre-MGB**	**Post-MGB**	***P*-value**
N (male/female)	20 (5/15)	20 (5/15)	
Diabetic/non-diabetic	20 (4/16)	20 (1/19)	
BMI (kg/m^2^)	43.33 ± 5.53	39.77 ± 5.13	<0.001
FBS (fasting blood glucose) (mg/dl)	106.04 ± 21.55	93.54 ± 20.84	0.004
HOMA2 IR	2.35 ± 0.67	1.95 ± 0.54	0.019
HOMA2 %B	132.44 ± 47.38	150.16 ± 41.74	0.041
DPP4 activity (nmol/min/ml)	15.66 ± 6.16	17.55 ± 4.51	0.084
DPP4 concentration (μg/ml)	64.99 ± 24.56	74.78 ± 21.17	0.163

*Data represented by means ± SD. p ≤ 0.05 considered statistically significant. Pre-MGB refers to the status before Mini Gastric Bypass and Post-MGB 4–6 weeks after Mini Gastric Bypass*.

## Discussion

Combining both the studies we show that plasma DPP4 activity is significantly increased in T2DM population irrespective of obesity pointing out to the fact that adipose-derived DPP4 has no major contribution on plasma DPP4 activity. Interestingly plasma DPP4 level was increased in the obese T2DM group compared to the non-obese T2DM group putatively due to the contribution of adipose tissue in determining the plasma DPP4 level. However, this increase in plasma DPP4 level in the obese T2DM group had no effect on the plasma DPP4 activity as plasma DPP4 activity was comparable between obese and non-obese T2DM groups. DPP4, being an adipokine, has been suggested to be a connecting link between adiposity and T2DM (19). However, consequent meta-analysis reported DPP4 inhibitors to exhibit greater glucose-lowering capacity in Asians with predominantly non-obese population demanding further investigation of DPP4 in relation to BMI (17, 18). The role of DPP4 in relation to BMI and T2DM was investigated in the experiments with SAT and VAT depots collected only from the obese subjects where we show that adipose tissue derived DPP4 activity (Figure 1) was similar between T2DM and non-T2DM subjects. Lastly, the prospective study with patients undergoing MGB surgery clearly point out to the fact that weight loss which is majorly a reduction in adipose tissue mass does not accompany a reduction in plasma DPP4 activity. Though a reduction of DPP4 activity with a concurrent increase in peak incretin levels was reported in patients after gastric bypass surgery, the authors found no correlation between increased incretin levels and DPP4 activity concluding that reduction in DPP4 activity occurs by a mechanism independent of weight loss (30). Consistently our results also showed DPP4 activity not to be associated with weight loss following bariatric surgery.

Our results are broadly in agreement with earlier studies exploring the possible association between plasma DPP4 activity and glycemic control (10). In a prospective study from China, DPP4 activity was suggested to be a predictor for the onset of insulin resistance, prediabetes, and T2DM independent of BMI (13). These observations indicate adipose derived DPP4 could contribute to its enhanced plasma levels but not DPP4 activity in obesity. Our data on obese patients having higher plasma DPP4 levels, a finding consistent with previously published reports (20, 22), with no significant change in plasma DPP4 activity suggest that increased DPP4 concentrations could not considerably contribute to DPP4 activity in obese population. Importantly in our study, plasma DPP4 levels and activity showed a modest correlation to each other in non-obese T2DM, but not in obese T2DM patients. Increased DPP4 secretion from adipose depot therefore may not significantly contribute to the DPP4 activity in the obese population. Although the mechanisms for such disconnect (20) between plasma DPP4 levels and activity in obese subjects are still not clear, our data collectively suggest that adipose tissue derived DPP4 does not significantly contribute to the active pool of plasma DPP4 activity.

DPP4 is ubiquitously expressed and shed into circulation, however system level information on the sourcing of tissue specific active form of DPP4 yet remains unknown. We have earlier shown that circulating Th17 cells shed DPP4 into the soluble pool through KLK5 dependent manner in T2DM (3). A recent mouse genetic study in adipose specific DPP4 knockout revealed non-essential role of adipose derived DPP4 for circulatory DPP4 activity, GLP-1 levels and glucose homeostasis, thus corroborating with our clinical data (31). Another elegant mouse genetic study highlights the role of endothelium derived DPP4 in GLP-1 degradation and glycemic control (32). Moreover, structural and biochemical evidence suggest that catalytically active DPP4 exists as higher order homodimeric or homotetrameric quaternary state (25, 26). Hence, discerning the tissue specific DPP4 production, its structural heterogeneity and its pathophysiological roles in T2DM require further investigation.

One important limitation for this study is the cut-off value for normal BMI. BMI being the crux of all these analyses, existing controversy surrounding the acceptance of BMI as criteria for identifying metabolically unhealthy non-obese patients calls for further debate (33, 34). Lack of those consensus criteria for discriminating non-obese from obese patients is a valid limitation for this study.

In summary, plasma DPP4 activity in T2DM is enhanced independent of BMI and adipose derived DPP4 is having no major role in glycemic regulation. Thus, plasma DPP4 activity in T2DM serves as an obesity independent parameter.

## Data Availability

The datasets generated for this study are available on request to the corresponding author.

## Ethics Statement

This study was carried out in accordance with the recommendations of Human Ethics Committee of Institute of Postgraduate Medical Education and Research (IPGME&R), Kolkata with written informed consent from all subjects. All subjects gave written informed consent in accordance with the Declaration of Helsinki.

## Author Contributions

JS and TN did the patient sampling, performed most of the experiments and analyzed data. OT and SG conducted the clinical evaluation and stratification of recruited patients. PC is the guarantor of this work and has full access to all the data in the study and takes responsibility for the integrity of the data and the accuracy of the data analysis, contributed to the study concept and design, data analysis, and writing of the manuscript.

### Conflict of Interest Statement

The authors declare that the research was conducted in the absence of any commercial or financial relationships that could be construed as a potential conflict of interest.
