# Supplementary material for: Increased Plasma Dipeptidyl Peptidase-4 (DPP4) Activity Is an Obesity-Independent Parameter for Glycemic Deregulation in Type 2 Diabetes Patients
Source: Front Endocrinol (Lausanne). 2019 Jul 25;10:505. doi: 10.3389/fendo.2019.00505 (PMC6670725; doi:10.3389/fendo.2019.00505)
Supplement: Supplementary file 1 [file Data_Sheet_1.docx]

**Supplementary Information**

**Increased Plasma Dipeptidyl Peptidase-4 (DPP4) Activity is an Obesity-independent Parameter for Glycemic Deregulation in Type 2 Diabetes Patients**

Jit Sarkar^1,2,3#^, Titli Nargis^1#^, Om Tantia^4^, Sujoy Ghosh^5^, Partha Chakrabarti^1,2*^

^1^Division of Cell Biology and Physiology, Indian Institute of Chemical Biology (CSIR), Kolkata, India

^2^Academy of Innovative and Scientific Research, Ghaziabad, India

^3^Community Health Program, SWANIRVAR, North 24 Parganas, West Bengal, India

^4^Department of Minimal Access & Bariatric Surgery, ILS Hospitals, Kolkata, India

^5^Department of Endocrinology and Metabolism, Institute of Postgraduate Medical Education and Research, Kolkata, India

^#^These authors have equal contributions

**Supplementary Figure 1.**

**Supplementary Figure 1. Plasma DPP4 concentrations and activity in obese and non-obese T2DM patients.** A-B. Boxplots showing plasma DPP4 concentrations (A) and DPP4 activity (B) between T2DM and healthy individuals. C-D. Plasma DPP4 concentration (C) and DPP4 activity within T2DM group grouped by BMI (D). E-F. Correlation between plasma DPP4 concentration and activity in non-obese (BMI ≤ 25) (E) and obese (BMI > 25) (F) T2DM patients. The boxplots represent the values as means ± SEM, pearson’s correlation coefficient expressed as ‘r’. P < 0.05 was considered statistically significant. p-values calculated by two-tailed test for boxplots (A-D) and by one-tailed test for correlation plots (E-F). 95% confidence interval plotted by dotted lines in correlation plots.

**Supplementary Figure 2.**

**Supplementary Figure 2. Four to six weeks follow up of plasma DPP4 concentrations and activity in obese patients underwent mini gastric bypass surgery.** Lineplots showing change in body mass index (A), fasting blood glucose (B), DPP4 Activity (C) and DPP4 concentration (D) before and after Mini Gastric Bypass bariatric surgery. *All panels*: two-tailed t-test was performed with *p<0.05.

**Supplementary Table 1. Correlation between all the parameters in non-obese and obese T2DM groups .**

| **Non-obese T2DM** | **Age (years)** | **BMI (kg/m**^2^) | **WC   (cms)** | **FBS (mg/dl)** | **HOMA2 IR** | **HOMA2 %B** | **TG (mg/dl)** | **TC (mg/dl)** | **DPP4-a** | **DPP4-c** |
| --- | --- | --- | --- | --- | --- | --- | --- | --- | --- | --- |
| **Age (years)** | 1, NA | 0.08,0.243 | 0.21,0.059 | 0.01,0.482 | -0.23,0.027 | -0.26,0.014 | -0.12,0.169 | -0.31,0.005 | -0.1,0.215 | -0.17,0.102 |
| **BMI (kg/m**^2^) |  | 1, NA | 0.77,0 | -0.12,0.152 | 0.19,0.06 | 0.08,0.256 | -0.13,0.157 | -0.05,0.346 | -0.18,0.075 | -0.1,0.234 |
| **WC (cms)** |  |  | 1, NA | -0.03,0.401 | 0.15,0.142 | 0.14,0.146 | -0.07,0.312 | -0.13,0.184 | -0.22,0.064 | -0.37,0.008 |
| **FBS (mg/dl)** |  |  |  | 1, NA | -0.46,0 | -0.03,0.414 | 0.27,0.013 | 0.28,0.011 | -0.04,0.364 | -0.01,0.458 |
| **HOMA2 IR** |  |  |  |  | 1, NA | 0.83,0 | -0.19,0.062 | -0.13,0.146 | -0.16,0.094 | -0.01,0.462 |
| **HOMA2 %B** |  |  |  |  |  | 1, NA | -0.08,0.265 | -0.04,0.382 | -0.13,0.145 | -0.05,0.355 |
| **TG (mg/dl)** |  |  |  |  |  |  | 1, NA | 0.42,0.001 | -0.18,0.079 | -0.07,0.313 |
| **TC (mg/dl)** |  |  |  |  |  |  |  | 1, NA | -0.12,0.171 | 0.28,0.021 |
| **DPP4-a** |  |  |  |  |  |  |  |  | 1, NA | 0.24,0.037 |
| **DPP4-c** |  |  |  |  |  |  |  |  |  | 1, NA |
|  |  |  |  |  |  |  |  |  |  |  |
| **Obese T2DM** | **Age (years)** | **BMI (kg/m**^2^) | **WC  (cms)** | **FBS (mg/dl)** | **HOMA2 IR** | **HOMA2 %B** | **TG (mg/dl)** | **TC (mg/dl)** | **DPP4-a** | **DPP4-c** |
| **Age (years)** | 1, NA | -0.07,0.31 | 0.03,0.437 | -0.21,0.065 | 0.05,0.365 | 0.07,0.322 | -0.3,0.022 | 0.01,0.463 | -0.08,0.293 | -0.34,0.035 |
| **BMI (kg/m**^2^) |  | 1, NA | 0.93,0 | -0.11,0.213 | 0.27,0.028 | 0.23,0.052 | -0.12,0.215 | -0.33,0.013 | -0.17,0.114 | 0.04,0.426 |
| **WC (cms)** |  |  | 1, NA | -0.26,0.059 | 0.52,0.001 | 0.29,0.039 | -0.09,0.296 | -0.35,0.017 | -0.08,0.307 | 0.11,0.325 |
| **FBS (mg/dl)** |  |  |  | 1, NA | -0.56,0 | -0.13,0.176 | 0.15,0.164 | 0.34,0.011 | 0.05,0.355 | -0.26,0.084 |
| **HOMA2 IR** |  |  |  |  | 1, NA | 0.74,0 | 0.05,0.377 | -0.27,0.033 | 0.07,0.304 | 0.09,0.321 |
| **HOMA2 %B** |  |  |  |  |  | 1, NA | 0.12,0.212 | -0.04,0.405 | 0.02,0.448 | 0.11,0.281 |
| **TG (mg/dl)** |  |  |  |  |  |  | 1, NA | 0.56,0 | -0.16,0.142 | 0.27,0.084 |
| **TC (mg/dl)** |  |  |  |  |  |  |  | 1, NA | -0.06,0.338 | 0.18,0.179 |
| **DPP4-a** |  |  |  |  |  |  |  |  | 1, NA | 0.13,0.258 |
| **DPP4-c** |  |  |  |  |  |  |  |  |  | 1,NA |

BMI: Body Mass Index; WC: Waist Circumference; FBS: Fasting Blood Glucose; TG: Triglycerides; TC: Total Cholesterol; DPP4-a: DPP4 activity (nmol/min/ml); DPP4-c: DPP4 concentration (µg/ml). Pearson’s correlation coefficient expressed as ‘r’. P < 0.05 was considered statistically significant. p-values calculated by one-tailed test and mentioned by comma separation after the correlation coefficient.
